# Persistence and Adherence to Biologics in Patients with Psoriasis in Taiwan: A New Biologics User Cohort Study

**DOI:** 10.3389/fphar.2022.880985

**Published:** 2022-05-17

**Authors:** Yu-Huei Huang, Chao-Hsiun Tang, Choo Hua Goh, Chia-Li Chang, Hong Qiu, Ya-Wen Yang, Carine Saadoun, Chia-Ling Chang, Yanfang Liu

**Affiliations:** ^1^ Chang Gung Memorial Hospital, Linkou, Division of Dermatology, and School of Medicine, Chang Gung University, Taoyuan, Taiwan; ^2^ School of Health Care Administration, Taipei Medical University, Taipei, Taiwan; ^3^ Epidemiology, Office of the Chief Medical Officer, Johnson & Johnson, Singapore, Singapore; ^4^ Epidemiology, Office of the Chief Medical Officer, Johnson & Johnson, Titusville, NJ, United States; ^5^ Immunology, Global Medical Affairs, Janssen Pharmaceutical Companies of Johnson & Johnson, Horsham, PA, United States; ^6^ Regional Medical Affairs, Janssen Asia-Pacific, Singapore; ^7^ Regional Medical Affairs, Janssen, Taiwan

**Keywords:** psoriasis, ustekinumab, persistence, adherence, cohort study

## Abstract

**Background:** Biologics are used to treat moderate-to-severe psoriasis, and persistence to biologics may reflect clinical effectiveness. Limited information describing how biologics are used in patients with moderate-to-severe psoriasis in Asian countries is available. We conducted a population-based, retrospective, new user cohort study using the National Health Insurance Research Database (NHIRD) in Taiwan to assess treatment persistence and adherence to biologics.

**Methods:** Adults with a diagnosis of psoriasis between 01 January 2015 and 31 December 2017 were identified in the NHIRD (ICD-9-CM 696.1; ICD-10 L40.0). New users were patients who initiated treatment with etanercept, adalimumab, ustekinumab or secukinumab between 01 January 2015 and 31 December 2017. All eligible patients were followed until 31 December 2018, death or disenrollment. Kaplan-Meier analysis was conducted to estimate persistence of treatment for index biologics. A Cox-proportional hazard regression model was used to compare risks of biologic discontinuation between biologic groups. Adjustments for potential confounding factors (age, gender and Charlson comorbidity index score) were made in the Cox model.

**Results:** There were 1,397 new biologic users with psoriasis during the study period. The ratio men:women was approximately 4:1. Mean age of patients ranged from 44.6 to 47.7 years across exposure groups. The 1-year/2-years persistence rates were 94.2%/84.9% for ustekinumab, 96.2%/not calculated (due to too few patients at year 2) for secukinumab, 66.0%/29.9% for etanercept, and 59.8%/40.3% for adalimumab. The risk of discontinuation was significantly lower in patients initiating ustekinumab compared with adalimumab (hazard ratio adjusted for age, sex and co-morbidities 0.289, 95%CI 0.247–0.339, *p* < 0.0001). Drug survival was significantly higher for ustekinumab compared with adalimumab and etanercept (log-rank test *p* < 0.0001). The proportions of patients with 1-year/2-years medication possession ratios of ≥80% were 95.3%/92.0% for ustekinumab, 98.1%/not calculated for secukinumab, 89.4%/83.1% for etanercept, and 70.8%/59.4% for adalimumab.

**Limitations:** Clinical improvement and response to treatment data were not available.

**Conclusion:** There was relatively high persistence amongst biologic users with psoriasis in Taiwan. There is a trend towards greater persistence of ustekinumab compared to other biologics, the magnitude of which depends on the treatment gap used for its calculation. This study provides real-world evidence that may facilitate optimal treatment choice.

## 1 Introduction

Psoriasis is a chronic condition of immune dysregulation believed to be caused by environmental triggers in genetically predisposed individuals (Das et al., 2019). The epidemiology of psoriasis varies geographically and disease prevalence is lower in Asian populations than in Caucasian populations (Chiu et al., 2018). In Taiwan, for example, the prevalence of psoriasis is 0.24% versus 2–11% in Caucasians (Chiu et al., 2018). In Asian patients the prevalence among males exceeds that of females by approximately 20% (ratio of 1.23:1), and men are more likely than women to have severe disease (Chang et al., 2009). Biologic drugs, such as monoclonal antibodies and fusion proteins that target specific components of the immune system have revolutionized treatment of moderate-to-severe psoriasis (Olszewska et al., 2018). The main drawbacks of these treatments are their high cost, and the occurrence of initial or secondary lack of efficacy, both of which represent common reasons for discontinuation (Wang et al., 2014; Youn et al., 2016).

Persistence is defined as the period of time from treatment initiation to discontinuation and is widely considered to be a marker of treatment success for drugs used to treat chronic diseases (Catalan and LeLorier 2000; Cramer et al., 2005; Yu et al., 2005; Costanzo et al., 2018). In real-world settings, longer persistence of biologic treatment in patients with psoriasis was associated with better effectiveness, tolerability, and patient satisfaction (Gniadecki et al., 2011; Gniadecki et al., 2015; Lories and de Vlam 2014). Conversely, treatment discontinuation may imply intolerability, adverse effects or futility with therapy. This is especially the case in Taiwan where continued reimbursement of the cost of a biologic treatment is provided only with evidence of clinical improvement (a 50% reduction in the psoriatic area and severity index score [PASI50]), as evaluated by a dermatologist every 6 months, and for longer than 2 years if the PASI50 criterion is met and the absolute PASI remains >10 indicating ongoing clinically significant disease. The consensus statement from the Taiwanese Dermatological Association notes that PASI75 (a 75% reduction in PASI) is used to evaluate treatment success. However, in Taiwan PASI50 coupled with improved quality of life is still acceptable to continue treatment (Tsai et al., 2017). The National Health Insurance (NHI) in Taiwan reimburses treatment with biologics for patients with psoriasis who meet eligibility criteria, including prior failure with phototherapy and at least two conventional systemic agents such as methotrexate, acitretin, or cyclosporin. In Taiwan, the tumor necrosis factor-inhibitors adalimumab and etanercept, the anti-interleukin-12/23 biologic ustekinumab, and the interleukin-17A inhibitors secukinumab and ixekizumab, are approved for reimbursement when used for the treatment of moderate-to-severe psoriasis. Reimbursement may continue indefinitely if improvement is sustained. Fewer than 2% of patients with psoriasis used biologics in 2013 in Taiwan (Wang, et al., 2016), and real-world information describing how biologics are used is lacking. To address this data gap, we conducted a retrospective cohort study utilizing the population-based NHI Research Database (NHIRD) in Taiwan. We examined the characteristics of patients with psoriasis who were prescribed biologics and assessed treatment persistence and drug adherence.

## 2 Methods

### 2.1 Study Design and Population

We conducted a retrospective, new user cohort study using the NHIRD database in Taiwan over the period from 01 January 2014 to 31 December 2018. The index date was the date of the first biologic prescription during the enrolment period. The baseline period was defined as the 12-month period prior to the index date. The Charlson-comorbidity index (CCI) score was calculated for all patients for the baseline period. All eligible patients were followed until the end of the observation period (31 December 2018), death or disenrollment from the NHIRD.

Reimbursement by the NHI for the treatment of moderate-to-severe psoriasis in Taiwan commenced on 1 November 2009 for etanercept, 01 July 2011 for adalimumab, 01 May 2012 for ustekinumab, 01 September 2016 for secukinumab, and 01 August 2018 for ixekizumab. Since ixekizumab was approved near the end of our study period, the absence of a sufficient follow-up period (at least 1 year) precluded its inclusion in this study.

Patients with a diagnosis of psoriasis vulgaris (International Classification of Diseases, Ninth Revision, Clinical Modification [ICD-9-CM)] 696.1 and ICD-10 code L40.0) between 01 January 2015 and 31 December 2017 were identified in the NHIRD. The diagnosis was considered confirmed if patients had at least either one inpatient claim or two consecutive outpatient claims for psoriasis during the observational period. New biologic users were patients who had at least one diagnosis of psoriasis, had not used any biologic treatment previously (bio-naïve), and initiated a biologic treatment between 01 January 2015 and 31 December 2017.

Eligible patients were new users of biologics ≥18 years of age who had data available in the NHIRD over a baseline period of ≥12 months prior to the index date. Patients were excluded if they had a labelled contraindication for biologic treatment (including prior diagnosis of malignancy; active hepatitis B or C; or receiving treatment for active tuberculosis) before the index date. Patients were also excluded if were using the biologics for other approved autoimmune indications other than psoriasis.

### 2.2 Data Source

The NHI has provided mandatory universal health insurance coverage for 99% of the Taiwanese population (approximately 23 million persons) since 1995 (National Health Insurance Research Database, 2013). The Ministry of Health and Welfare maintains the Health and Welfare Data Science Center, which houses the longitudinal, population-level claims-based NHIRD for all of Taiwan (Hsieh et al., 2019). The NHIRD holds information on all medical services provided to the Taiwanese population. The database captures demographic information, all medical diagnoses in ICD-9-CM through 2015 and ICD-10 format from 2016 onward, and all inpatient and outpatient episodes, prescriptions, pharmacy treatments, and other services, including imaging and laboratory examinations.

### 2.3 Variables and Outcomes

Patient demographic and clinical characteristics including age, sex, and CCI were assessed for the baseline period.

Persistence was defined as continuous treatment with the biologic without the presence of a pre-defined treatment gap. There is no standard definition of a treatment gap; therefore, we defined the treatment gap according to the maintenance dosing administration schedule, which is weekly for etanercept, every 2 weeks for adalimumab, every 12 weeks for ustekinumab, and every 4 weeks for secukinumab (Table 1). Accordingly, the treatment gap was defined as approximately twice the maintenance dosing interval for each biologic; 15 days for etanercept, 30 days for adalimumab, 180 days for ustekinumab, and 60 days for secukinumab, starting after the last day of supply of the last prescription.

**TABLE 1:** Administration regimen of biologics reimbursed for the treatment of psoriasis in Taiwan, and treatment gap.

Biologic	Induction Dose	Maintenance Dose	Dose Interval	Treatment Gap
Adalimumab	80 mg single dose	40 mg starting 1 week after the induction dose	Every 2 weeks	30 days
Etanercept	50 mg twice weekly for 12 weeks	50 mg *or*	Weekly	15 days
		25 mg	Twice weekly	
Ustekinumab	45 mg at 0 and 4 weeks	45 mg	Every 12 weeks	180 days
Secukinumab	300 mg every week for 5 doses	300 mg (150 mg if ≤ 60 kg)	Every 4 weeks	60 days

Treatment was considered to have been discontinued if a period exceeding the defined treatment gap with no detected drug supply was identified. The date of discontinuation was the date of the last supply of the biologic agent plus the duration of the last prescription and the treatment gap. Drug survival was measured in consecutive days, from the date of the first biologic prescription until discontinuation, switching (a prescription for a different biologic during the treatment gap), or study end, whichever occurred first.

Compliance was expressed as the medication possession ratio (MPR) which is the proportion of days of medication supply during the period on therapy. The MPR for each patient in each biologic group was calculated as:

$$\frac{Total\;number\;of\;drug\;days\;prescribed}{Last\;prescription\;date-first\;prescription\;date+last\;prescribed\;day\;of\;supply\;}$$

### 2.4 Statistical Analysis

Patient baseline characteristics were presented descriptively using mean (standard deviation [SD]), median and interquartile range (IQR) for continuous variables, and frequency and percentage for categorical variables. Differences between treatment groups were evaluated using t-tests for continuous variables and chi-square tests for categorical variables. A *p*-value of <0.05 was considered statistically significant.

Persistence was calculated as the proportion of patients remaining on the index biologic after the index date and was assessed at both 1 and 2 years. The MPR was calculated for both 1 and 2 years after the index date using a threshold of 80% to indicate compliance. Secukinumab was excluded from year 2 analyses because of insufficient data at 2 years of follow up. Sensitivity analyses using treatment gaps of 45 and 90 days were used to validate the results.

Drug survival was calculated as the percentage of patients remaining on the index biologic using the Kaplan-Meier method and the log-rank test was used to compare survival between the different exposure groups. Cox-proportional hazard regression was used to determine the adjusted hazard ratio (aHR) for comparing rates of biologic discontinuation between each exposure group and the adalimumab group, with adjustment for age, gender and CCI score. Secukinumab was not included in the analysis for discontinuation given the small number of users.

The exact method was used to compute 95% confidence intervals (CI). Kaplan-Meier analysis was performed using STATA version 15.0 (StataCorp, College Station, TX). All other analyses were performed using SAS, Version 9.4 (Cary, NC, United States).

### 2.5 Ethics Approval Statement

The analysis used de-identified aggregated patient data and was conducted in accordance with all applicable guidelines and regulations. The study was granted an exemption from ethical review and an exemption from the need for patient consent by the Taipei Medical University-Joint Institutional Review Board.

## 3 Results

**FIGURE 1:**
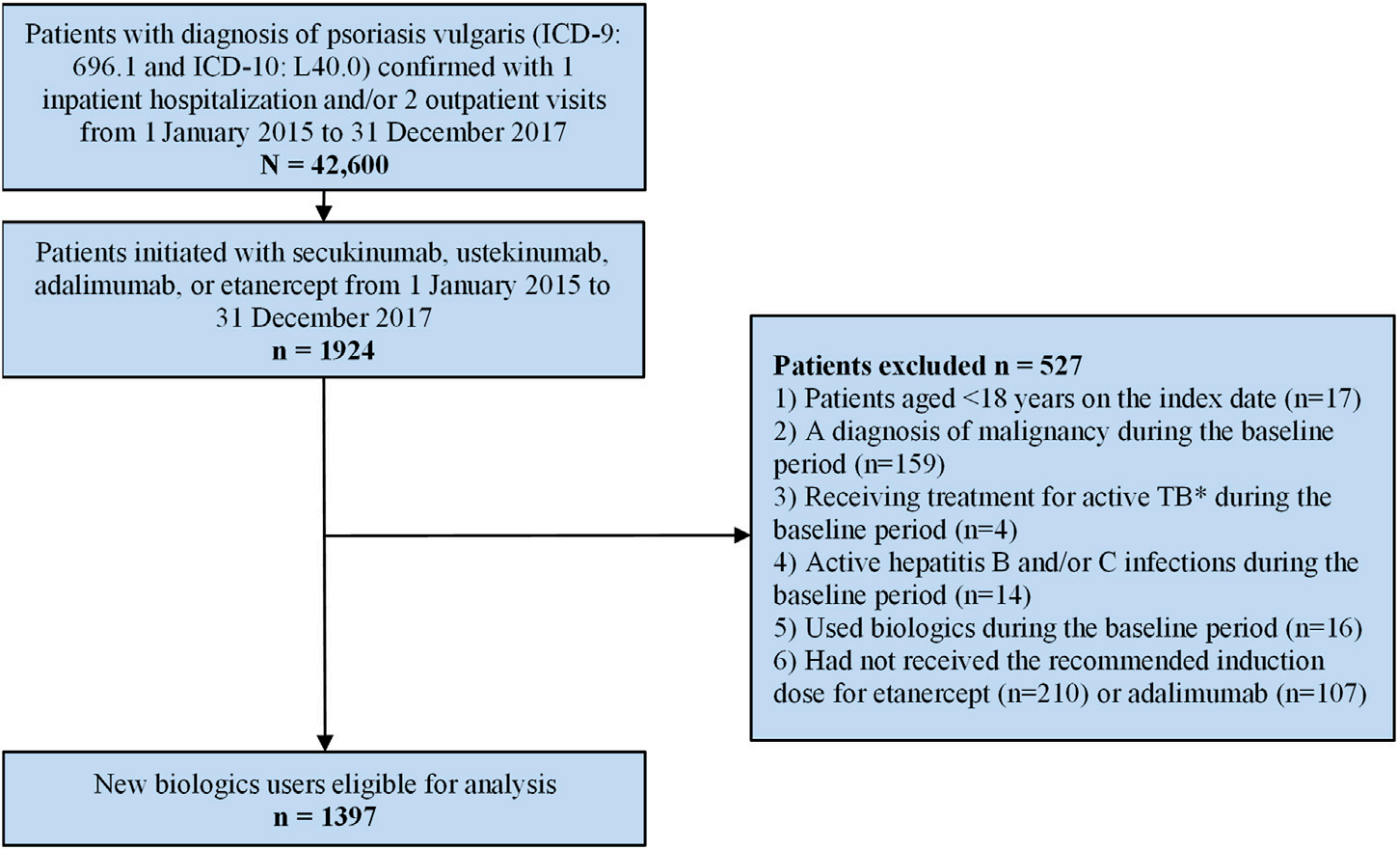
Patient enrolment flow-chart. * Active TB is defined as having a diagnosis of tuberculosis and claims for at least two anti-TB medications over a 6-month period.

A total of 42,600 patients with a diagnosis of psoriasis vulgaris based on the inclusion criteria for this study were identified in the NHIRD between 1 January 2015 and 31 December 2017. Of these, 27,459 were men and 14,711 were women (unknown for 430, male to female ratio 1.87). Among the identified patients with psoriasis there were 1924 who were new users of biologics (Figure 1). After applying exclusion criteria, 1,397 patients were included in the cohort analysis; 824 patients were new users of ustekinumab, 52 were new users of secukinumab, 94 were new users of etanercept, and 427 were new users of adalimumab (Table 2).

**TABLE 2:** Baseline characteristics of new users of biologics for treatment of psoriasis (*N* = 1,397).

Variable	Ustekinumab	Secukinumab	Etanercept	Adalimumab
	*N* = 824	*N* = 52	*N* = 94	*N* = 427
	n (%)	n (%)	n (%)	n (%)
Gender				
Male	645 (78.3)	44 (84.6)	71 (75.5)	301 (70.5)
Female	179 (21.7)	8 (15.4)	23 (24.5)	126 (29.5)
Age on biologic index date, years				
Median (IQR)	45 (35.0–56.0)	43.5 (34.0–57.5)	48 (38.0–58.0)	44.0 (35.0–54.0)
Range	18–84	19–77	18–85	18–78
18–29	102 (12.4)	7 (13.5)	4 (4.3)	40 (9.4)
30–39	191 (23.2)	12 (23.1)	23 (24.5)	128 (30)
40–49	191 (23.2)	14 (26.9)	24 (25.5)	117 (27.4)
50–59	201 (24.4)	8 (15.4)	30 (31.9)	86 (20.1)
60–69	107 (13)	8 (15.4)	10 (10.6)	44 (10.3)
≥70	32 (3.8)	3 (5.7)	3 (3.2)	12 (2.8)
CCI				
Mean ± SD	0.7 ± 1.2	0.7 ± 0.9	0.9 ± 1.3	0.8 ± 1.2
Median (IQR)	0 (0–1)	0 (0–1)	0 (0–1)	0 (0–1)
CCI = 0	510 (61.9)	29 (55.8)	51 (54.3)	240 (56.2)
CCI = 1	181 (22)	13 (25)	20 (21.3)	120 (28.1)
CCI = 2	65 (7.9)	7 (13.5)	9 (9.6)	29 (6.8)
CCI ≥3	68 (8.3)	3 (5.8)	14 (14.9)	38 (8.9)
CCI				
Diabetes without chronic complication	119 (14.4)	4 (7.7)	15 (16)	54 (12.6)
Mild liver disease	112 (13.6)	10 (19.2)	11 (11.7)	55 (12.9)
Peptic ulcer disease	65 (7.9)	5 (9.6)	18 (19.1)	48 (11.2)
Chronic pulmonary disease	59 (7.2)	^a^	6 (6.4)	37 (8.7)
Renal disease	35 (4.2)	^a^	9 (9.6)	18 (4.2)
Cerebrovascular disease	33 (4)	^a^	^a^	11 (2.6)
Diabetes with chronic complication	31 (3.8)	^a^	4 (4.3)	15 (3.5)
Congestive heart failure	11 (1.3)	^a^	0	6 (1.4)
Rheumatic disease	11 (1.3)	0	8 (8.5)	35 (8.2)
Myocardial infarction	7 (0.8)	0	^a^	^a^
Peripheral vascular disease	4 (0.5)	0	^a^	0
Hemiplegia or paraplegia	3 (0.4)	0	0	^a^
Moderate or severe liver disease	3 (0.4)	0	0	^a^

CCI, charlson comorbidity index; IQR, inter-quartile range; N, number of patients; SD, standard deviation.

a All non-zero counts that were less than three were suppressed to protect patient privacy.

Men outnumbered women in each exposure group at a ratio of approximately 4:1. The proportion of men was significantly higher in the secukinumab (84.6%, *p* = 0.0322) and ustekinumab (78.3%, *p* = 0.0024) groups compared to the adalimumab group (70.5%) (Table 2).

The median age of patients was similar in the ustekinumab (45.0 years, IQR 35.0–56.0), secukinumab (43.5 years, IQR 34.0–57.5) and adalimumab (44.0 years, IQR 35.0–54.0) exposure groups, and was higher (48 years, IQR 38.0–58.0) in the etanercept group. The mean CCI scores were 0.7 in the ustekinumab and secukinumab groups, 0.9 in the etanercept group, and 0.8 in the adalimumab group. The distribution of co-morbidities was generally similar in each exposure group. The three most common co-morbidities in each group, although differing in prevalence across groups, were diabetes without chronic complication, mild liver disease, and peptic ulcer disease (Table 2).

The mean follow-up time per person was 2.60 years in the ustekinumab group, 1.55 years in the etanercept group, and 1.53 years in the adalimumab group.

### 3.1 Persistence and Drug Survival

There were 1,393 patients (99.7%) with at least 1 year of follow-up after the index date and 1,111 patients (79.5%) with at least 2 years of follow-up. The rates of persistence 1 year after the index date for new biologics users were 96.2% for secukinumab, 94.2% for ustekinumab, 66.0% for etanercept and 59.8% for adalimumab (Table 3).

**TABLE 3:** Persistence rates at 1 and 2 years after the index date in new users of biologics for the treatment of psoriasis.

Follow-Up Period	Ustekinumab	Secukinumab^a^	Etanercept	Adalimumab
Schedule-Based Treatment gap^b^				
1 year				
Persistence rate (95% CI)	94.2 (92.6–95.8)	96.2 (90.7–100)	66.0 (56.2–75.7)	59.8 (55.1–64.4)
2 years				
Persistence rate (95% CI)	84.9 (72.2–87.6)	NA	29.9 (19.4–40.3)	40.3 (35.1–45.5)
90-days treatment gap				
1 year				
Persistence rate (95% CI)	91.6 (89.7–93.5)	96.2 (90.7–100)	84.0 (76.5–91.6)	75.8 (71.7–79.9)
2 years				
Persistence rate (95% CI)	76.2 (73.0–79.4)	NA	62.3 (51.3–73.4)	62.6 (57.5–67.7)
45-days treatment gap				
1 year				
Persistence rate (95% CI)	88.0 (85.7–90.2)	94.2 (87.7–100)	77.7 (69.1–86.2)	68.5 (60.4–72.9)
2 years				
Persistence rate (95% CI)	68.8 (65.3–72.3)	NA	53.2 (41.8–64.6)	51.3 (46.0–56.6)

CI, confidence interval, NA: not applicable.

a secukinumab was not included at second year of analysis because of the low number of patients.

b 30 days gap for adalimumab, 15 days gap for etanercept, 180 days gap for ustekinumab, 60 days gap for secukinumab.

At year 2, persistence rates had declined for all exposure groups but was highest for the ustekinumab group. Persistence at year 2 was 40.3%in the adalimumab group and 29.9% in the etanercept group. Secukinumab was excluded from year 2 analyses because there were very few (8) patients with a 2 years of observation period.

Sensitivity analyses were conducted using 45-days and 90-days treatment gaps for all biologics regardless of the recommended administration schedule. While persistence rates for adalimumab and etanercept were higher under the longer treatment gaps, persistence of ustekinumab and secukinumab remained higher than adalimumab and etanercept under both scenarios (45-days gap: 88.2% at year 1 and 68.8% at year 2 for ustekinumab, and 94.2% at year 1 for secukinumab; 90-days gap: 91.6% at year 1 and 76.2% at year 2 for ustekinumab and 96.2% at year 1 for secukinumab) (Table 3).

Amongst all patients initiating biologic therapy, 709 (50.8%) discontinued or switched treatment during the study period. The incidence of discontinuation overall was 23.5 per 100 person-years, but varied between treatment groups; 15.0 per 100 person-years for ustekinumab users, 48.8 per 100 person-years for etanercept users, and 47.8 per 100 person-years for adalimumab users (Table 4). The percentage of patients who switched to another biologic during the study period was 14.8% (122/824) among ustekinumab users, 14.9% (14/94) among etanercept users, and 9.6% (41/427) among adalimumab users.

**TABLE 4:** Crude and adjusted hazard ratio of drug discontinuation (non-persistence) in new users of biologics for the treatment of psoriasis using a Cox proportional hazards model.

Biologic^a^	N	Events^b^	Person-years	Incidence Rate per 100 Person-years	Crude HR (95%CI)	*p*	Adjusted HR (95%CI)^c^	*p*
Ustekinumab	824	321	2146	15.0	0.292 (0.250–0.342)	<0.0001	0.289 (0.247–0.339)	<0.0001
Etanercept	94	71	145.4	48.8	1.061 (0.820–1.374)	0.6516	1.044 (0.806–1.352)	0.7438
Adalimumab	427	312	652.5	47.8	Ref = 1	-	Ref = 1	-

CI, confidence interval; HR, hazard ratio; N, number of patients.

a Secukinumab was not included in the analysis because of the low number of patients initiated on this drug.

b Discontinuation or switching.

c Adjusted for gender, age (continuous variable) and CCI (continuous variable).

The risk of treatment discontinuation was significantly lower for patients who initiated ustekinumab compared with adalimumab after adjusting for sex, age and CCI (aHR 0.289, 95% CI 0.247–0.339, *p* < 0.0001). There were no significant differences in drug discontinuation among patients who initiated etanercept compared to adalimumab in either the crude or adjusted analyses (Table 4).

**FIGURE 2:**
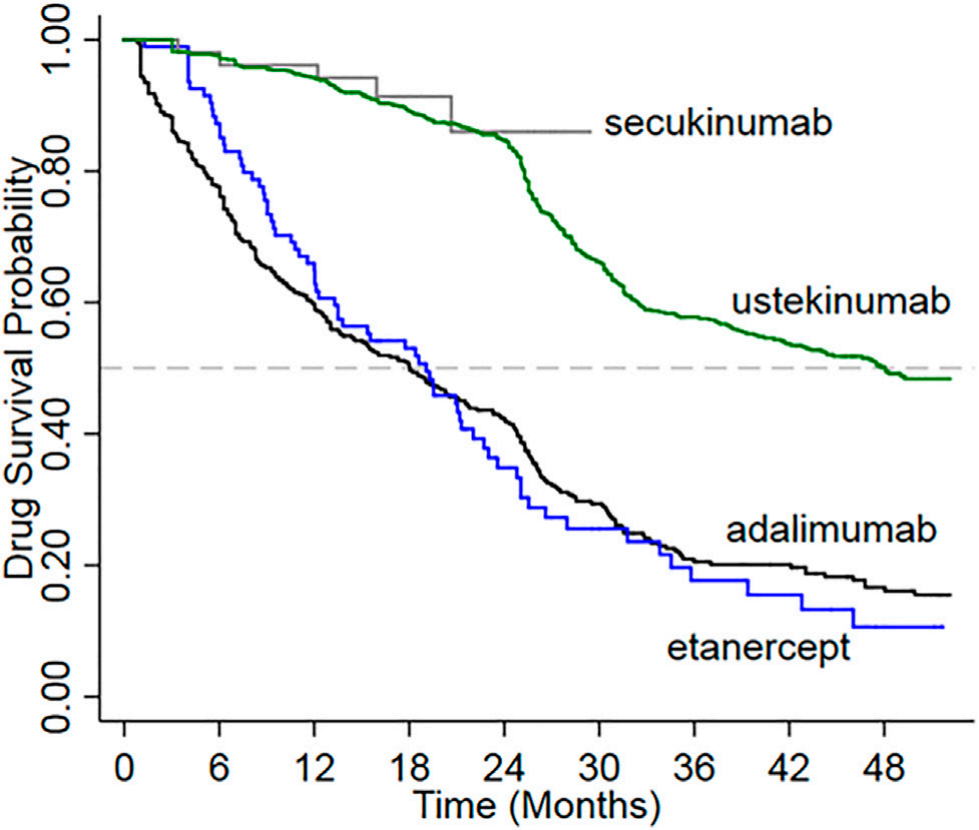
Drug survival among new users of biologic treatments for psoriasis.

Drug survival was significantly higher for new users of ustekinumab compared with adalimumab and etanercept (log-rank test *p* < 0.0001) (Figure 2). The mean duration of follow-up was shortest for the group treated with secukinumab, for which reimbursement commenced in 2016. Drug survival among new users of secukinumab appeared to be similar to that for ustekinumab over a comparable period of follow-up.

### 3.2 Medication Possession Ratio

At 1 year after the index date, the proportion of patients with a MPR of ≥80% was significantly higher for new users of ustekinumab (95.3%), secukinumab (98.1%), and etanercept (89.4%) compared to new users of adalimumab (70.8%, *p* ≤ 0.0002 for all comparisons) (Table 5). At year 2, the proportion of patients with a MPR of ≥80% was 92.0% for ustekinumab users, 83.1% for etanercept users, and 59.4% for adalimumab users. MPR was not calculated for the secukinumab group at year 2 given the small number of remaining patients. The proportion of patients with a MPR of ≥80% remained significantly higher for users of ustekinumab and etanercept compared to adalimumab at year 2 (*p* < 0.0001 for both comparisons).

**TABLE 5:** Proportion of patients with medication possession ratio (MPR) of at least 80% at 1 and 2 years after the index date in new users of biologics for the treatment of psoriasis.

Follow-Up Period	Ustekinumab	Secukinumab^a^	Etanercept	Adalimumab
1 year				
MPR ≥80% (95% CI)	95.3% (93.6–96.6)	98.1% (89.7–100)	89.4% (81.3–94.8)	70.8% (66.3–75.1)
*p*-value	<0.0001	<0.0001	0.0002	Reference
2 years				
MPR ≥80% (95% CI)	92.0% (89.7–93.9)	NA	83.1% (72.9–90.7)	59.4% (54.0–64.7)
*p*-value	<0.0001	NA	<0.0001	Reference

CI, confidence interval.

a secukinumab was not included in the year 2 analysis because of the low number of patients.

## 4 Discussion

We used a comprehensive, longitudinal, population-based database that includes virtually all residents of Taiwan to investigate patterns of drug persistence and compliance in patients with moderate-to-severe psoriasis who were new users of biologics. We observed similarly high rates of persistence among users of secukinumab and ustekinumab 1 year after initiation. A high rate of persistence (84.9%) was sustained through 2 years after initiation of ustekinumab. However, too few secukinumab users were available for analysis at this time point. The persistence rate at year 2 was markedly lower among users of etanercept and adalimumab (29.9 and 40.3%, respectively at 2 years). The risk of discontinuation was 3.4-fold lower for patients who initiated ustekinumab compared to adalimumab. The median follow-up time was highest for ustekinumab, which suggests that patients tend to stay on ustekinumab for longer periods, possibly reflecting better effectiveness than the other biologics included in this analysis.

Compliance with biologic treatment, as measured by the proportion of patients with a MPR of ≥80%, was significantly higher for patients who initiated ustekinumab and etanercept compared to adalimumab at both year 1 and year 2. Despite etanercept displaying the lowest persistence rate observed, the proportion of patients with a MPR of ≥80% remained high among those who continued etanercept for up to 2 years (83.1%) and was significantly higher than that for those who continued adalimumab (59.4%). Of note, etanercept has the most frequent (weekly or twice weekly) dosing regimen of those studied. The high degree of compliance could potentially reflect lower effectiveness for etanercept, in that consistently regular administration may be needed to maintain clinical benefit, whereas patients receiving adalimumab may be able to dose less frequently and maintain response.

To our knowledge, this is the first study to evaluate treatment patterns of biologics such as ustekinumab and secukinumab in patients with psoriasis in an Asian country. The results are particularly pertinent for Taiwan, where the reimbursement environment for biologics is linked to periodic (6 months interval) demonstration of clinical effectiveness (at least PASI50 compared with baseline). In this setting, persistence can be considered as a proxy of clinical improvement. Of note, unlike real-world studies conducted in other countries (Sbidian et al., 2019), male new users of biologics outnumbered women by 2.4 to 5.5-fold with the highest proportions of men in the secukinumab (5.5-fold more males than females) and ustekinumab (3.6-fold more males than females) groups. This discrepancy likely reflects the higher prevalence of severe psoriasis among males than females in Taiwan (Chang et al., 2009), although other reasons for prescribing biologics more often for men than women cannot be excluded.

We identified only 5 studies that previously reported persistence data for at least ustekinumab among new biologics users with psoriasis in which patients with psoriatic arthritis or other autoimmune diseases were excluded (Howe et al., 2014; Feldman et al., 2015; Chastek et al., 2016; Sauer et al., 2016; Pina Vegas et al., 2022) (Table 6). One study (Howe et al., 2014) included only 7 patients who received ustekinumab and was not considered further. Among the other 4 studies, two defined the treatment gap as 45 days for all biologics, one defined the treatment gap as 60 days, and one used a different gap for each biologic based on the dosing interval.

**TABLE 6:** Summary of published studies of drug persistence using claims databases in new users of biologics for the treatment of psoriasis^a^.

Country (Reference)	Study years	Data Source	N of Patients or Treatment Episodes	Treatment Gap	Persistence at 1 year		
					Ustekinumab	Adalimumab	Etanercept
US Chastek, et al. (2016)	2011–2013	Optum Research Database	1992	45 days	33.2	34.5	31.4
US Sauer, et al. (2016)	2008–2011	Veterans’ Health Administration	369	45 days	52.6	50.8	46.7
US Feldman et al., (2015)	2007–2012	MarketScan Commercial Encounters Database	4309	4 weeks for etanercept, 8 weeks for adalimumab, 18 weeks for ustekinumab	70.8	53.4	19.0
France Pina Vegas et al. (2022)	2015–2019	French health insurance scheme	16,892	60 days	81.1	72.9	69.7

a Studies in new biologics users that excluded patients with psoriatic arthritis. The study by Howe et al. (Howe et al., 2014) only included 7 patients who received ustekinumab so is not considered in this table.

Log-rank test, *p* < 0.0001.

*All non-zero counts that were less than three were suppressed to protect patient privacy.

The studies that used a treatment gap definition of 45 days for all biologics (Chastek et al., 2016; Sauer et al., 2016), showed no or negligible differences in persistence between biologics at 1 year of follow up. Given that the recommended maintenance dosing interval for ustekinumab is every 12 weeks (84 days), a 45-days gap may not be sufficient to assess discontinuation of ustekinumab. The study that used a 60-days treatment gap reported results that are consistent with our own; 1-year persistence in patients with psoriasis was 81.1% for ustekinumab versus 72.9% for adalimumab and 69.7% for etanercept (Pina Vegas et al., 2022). Over the entire 3-years observation period ustekinumab was associated with higher persistence than all tumor necrosis factor inhibitors (HR 0.76, 95% CI 0.72–0.80) (Pina Vegas et al., 2022). Persistence was also substantially higher among ustekinumab users (70.8 versus 53.4% for adalimumab and 19.0% for etanercept) in the study that used a dose-defined treatment gap for each biologic (Feldman et al., 2015). Different dosing intervals between biologic drugs has been cited as a reason for caution in interpreting drug survival data (Sbidian, et al., 2019). In turn, use of the same treatment gap definition for drugs that differ markedly in their maintenance dosing interval may represent a limitation of some drug survival studies. There is no consensus on the most appropriate treatment gap used to define discontinuation of biologic drugs in patients with psoriasis.

Within the Asia-pacific region, PASI is routinely used as the response criterion for re-imbursement purposes, although the use of PASI50 and PASI75 varies (Tsai and Yeh 2012). Our 2-years estimate of persistence of 84.9% among ustekinumab users is higher than reported previously and could reflect the stringent criteria used to select patients for reimbursement of biologic treatment in Taiwan. Treatment is limited to patients who have moderate-to-severe disease and who have failed other conventional therapies. Moreover, cost is a major reason for drug discontinuation in Asian countries (Youn et al., 2016). We eliminated the impact of cost on treatment discontinuation by studying the period after reimbursement by the NHI was introduced for eligible patients. Re-imbursement for a biologic may continue beyond 2 years if the patient continues to meet the PASI50 response criterion and if they have ongoing evidence of disease based on an absolute PASI score >10. We observed a decrease in the rate of drug survival after 2 years for ustekinumab, which likely reflects patients with a sufficiently good response achieving an absolute PASI score <10.

Strengths of our study are the well-defined patient population, and the ability to capture through the NHIRD comprehensive and complete long-term data for patients receiving biologic therapy suitable for evaluating drug treatment patterns. The use of clinical eligibility criteria and regular monitoring of clinical effectiveness linked to reimbursement in Taiwan allows for measures of compliance such as persistence to be used as proxies for effectiveness and tolerability. The new user study design enabled following patients from the initiation of biologic treatment until discontinuation. We used a treatment gap based on the recommended maintenance dosing interval for each biologic agent to improve the comparability of persistence data.

A potential limitation of our study is that information about clinical response or reasons for switching/discontinuation could not be collected; therefore, assumptions regarding treatment effectiveness or safety considerations could not be confirmed. However, as clinical improvement is a criterion for continuing biologic treatment in Taiwan, persistent use may represent an indication of clinical effectiveness. On the other hand, patients who cease treatment due to clinical response or for other considerations such as surgery or infection and who do not relapse could not be identified. In our study, this could “penalize” drugs with more frequent administration schedules than ustekinumab. Although the NHIRD allows for complete capture of data regarding patient treatment, information on psoriasis treatments that were newly approved but not reimbursed by NHIRD, and complementary or traditional medications, was not captured. Furthermore, only 2.3% of patients with psoriasis in Taiwan were treated with a biologic during the study period, which limited the sample size of this study and the ability to perform comparative analyses. We excluded patients who received biologics for reasons other than psoriasis, and although we have attempted to adjust for confounders, possible residual confounding, such as smoking status, alcohol intake ad other lifestyle and environmental factors exsits (Zeng et al., 2017; Svanstrom et al., 2019; Zhou et al., 2020) could not be ruled out completely as this information is not recorded in the NHIRD. Other studies have reported a significant drop in persistence over time up until 3 years after treatment initiation (Pina Vegas et al., 2022), suggesting that longer follow-up of patients may be needed to more fully understand treatment patterns using biologics. Finally, while the study is representative of biologics use for patients with psoriasis in Taiwan, it may be less applicable to other settings with different healthcare systems.

In conclusion, we observed high rates of persistence and adherence amongst patient with psoriasis who initiated biologic therapy. There is a trend towards greater persistence at 1 and 2 years of ustekinumab compared to other biologics, the magnitude of which depends on the treatment gap used for its calculation. This study provides real-world evidence that may facilitate optimal treatment choice.

## Data Availability

The data underlying this study are from the NHIRD which has been transferred to the HWDC. The Taiwan government prohibits release of the NHI claims dataset to the public domain. Interested researchers can obtain the data through formal application to the HWDC, Department of Statistics, Ministry of Health and Welfare, Taiwan (http://dep.mohw.gov.tw/DOS/np-2497-113.html).
